# Dnmt3b ablation affects fracture repair process by regulating apoptosis

**DOI:** 10.1186/s12891-024-07283-7

**Published:** 2024-02-27

**Authors:** Xu Wang, Qinwen Ge, Qinghe Zeng, Kaiao Zou, Zhengsheng Bao, Jun Ying, Zhen Wu, Hongting Jin, Jiali Chen, Taotao Xu

**Affiliations:** 1 Institute of Orthopedics and Traumatology, the First Affiliated Hospital of Zhejiang Chinese Medical University (Zhejiang Provincial Hospital of Chinese Medicine), Hangzhou, Zhejiang Province China; 2 The First College of Clinical Medicine, Zhejiang Chinese Medical University, Hangzhou, Zhejiang Province China; 3 The Second College of Clinical Medicine, Zhejiang Chinese Medical University, Hangzhou, Zhejiang Province China; 4 Tongde Hospital of Zhejiang Province, Hangzhou, Zhejiang Province China

**Keywords:** Fracture healing, Periosteum stem cells, Dnmt3b

## Abstract

**Purpose:**

Previous studies have shown that DNA methyltransferase 3b (Dnmt3b) is the only Dnmt responsive to fracture repair and Dnmt3b ablation in Prx1-positive stem cells and chondrocyte cells both delayed fracture repair. Our study aims to explore the influence of Dnmt3b ablation in Gli1-positive stem cells in fracture healing mice and the underlying mechanism.

**Methods:**

We generated *Gli1-CreERT2; Dnmt3bflox/flox (Dnmt3b*^*Gli1ER*^*)* mice to operated tibia fracture. Fracture callus tissues of *Dnmt3b*^*Gli1ER*^ mice and control mice were collected and analyzed by X-ray, micro-CT, biomechanical testing, histopathology and TUNEL assay.

**Results:**

The cartilaginous callus significantly decrease in ablation of Dnmt3b in Gli1-positive stem cells during fracture repair. The chondrogenic and osteogenic indicators (Sox9 and Runx2) in the fracture healing tissues in *Dnmt3b*^*Gli1ER*^ mice much less than control mice. *Dnmt3b*^*Gli1ER*^ mice led to delayed bone callus remodeling and decreased biomechanical properties of the newly formed bone during fracture repair. Both the expressions of Caspase-3 and Caspase-8 were upregulated in *Dnmt3b*^*Gli1ER*^ mice as well as the expressions of BCL-2.

**Conclusions:**

Our study provides an evidence that Dnmt3b ablation Gli1-positive stem cells can affect fracture healing and lead to poor fracture healing by regulating apoptosis to decrease chondrocyte hypertrophic maturation.

## Introduction

Bones are found in many parts in our body which serve many functions such as support, locomotion, protection of soft tissues and it is a support structure for vertebrates [1]. In one human life, bones undergo continuous remodeling, repair, and regeneration [2]. Fractures can result in disability and increasing socioeconomic burden [3] which healing commonly uses within about 3 months. However, there are a lot of patients who can’t healing normally within the specified time because of various factors such as biological factors and technical factors [4].

Bone fracture injuries disrupt marrow sinusoidal architecture and surrounding soft tissues, and blood clots form in the fracture area that initiate an early local inflammatory process [5]. Traditional view is that the fracture healing has four stages, a new model has been put forward is that the fracture healing is a metabolic process. An anabolic phase involves chondrogenesis and osteogenesis and a catabolic phase involves remodeling of the cartilaginous and bony callus after anabolic phase [6]. So, bone remodeling and metabolic balance is essential for fracture healing.

At the site of injury, stem cells are recruited to the specific site to play it role. In the stage of fracture healing, a variety of stem cells are recruited to the fracture site, among which periosteal stem cells play a key role. In this period, periosteum stem cells (PSCs) which recruited from many sources proceed precisely regulated multidirectional differentiation involved in bone regeneration and vascularization [7–9].

The essential thing to obtain firm stability and rigidity of the fractured bones is that the process of cartilage-to-bone transition. This process failure or delay can cause an impaired bony union like nonunion or delayed union [10–12]. The cartilage-to-bone transition consists of multiple events such as the degradation of cartilaginous matrix, vascular invasion and bone formation. They are bidirectional and indispensable in fracture healing, and involve many cells including chondrocytes, osteoclasts, and bone cells. The hypertrophic chondrocytes have an intermediary role which between soft callus and hard callus [13].

In mammals, there is a central epigenomic regulation named as enzyme DNA methyltransferase (DNMT) which consists of three highly conserved proteins: DNMT1, DNMT3A, and DNMT3B [14–22]. Previous research has found that MSCs in fracture patients have differential methylation sites, which are related to their function, suggesting that DNA methylation mechanism driven by DNA methyltransferase (DNMTs) is involved in fracture healing [23]. In the recent findings, it provided evidences that Dnmt3b is widely expressed in chondrocytes and MSCs in callus region, first increases and then decreases in the process of fracture healing [24]. Moreover, when the Dnmt3b is ablated in chondrocytes that it can delayed fracture repair [25].

Our study explores the mechanism involved in the regulation of PSC proliferation and differentiation by Dnmt3b in both chondrogenic and osteogenic lineages during bone fracture healing. We show that the ablation of Dnmt3b is associated with bone remodeling and metabolic balance, leading to decreased cartilaginous and bony callus formation and impaired fracture repair in mice that is due to the abnormal apoptosis of chondrocytes. To sum up, our findings establish for the first time that Dnmt3b is crucial in the regulation of chondrocyte which differentiated from Gli1-positive stem cells apoptosis during endochondral bone repair process.

## Results

### Ablation of Dnmt3b in periosteum stem cells reduced cartilaginous callus formation and bony callus ossification during fracture repair

**Fig. 1:**
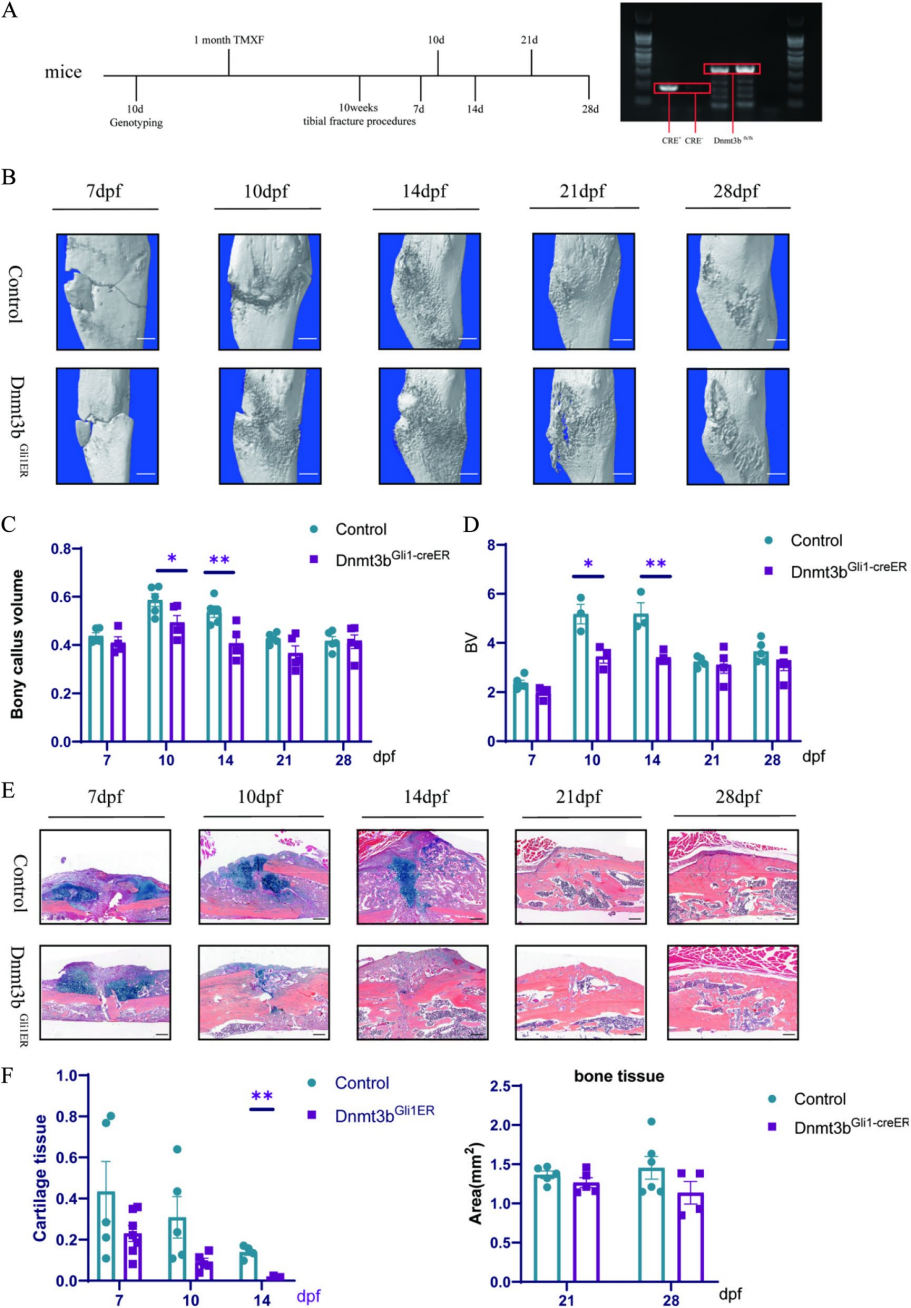
Ablation of Dnmt3b in periosteum stem cells reduced cartilaginous callus formation and bony callus ossification during fracture repair. **A** Experimental time node and mouse gene identification diagram. **B** Representative 3D Micro-CT reconstructions images of bone callus at 7, 10, 14, 21 and 28 days after fracture (dpf). Scale bar = 500 μm. **C** Quantitative analyses of the callus bone volume/total callus volume (callus bone volume%) in Dnmt3bGli1ER mice and control mice 7, 10 and 14dpf. **D** Quantitative analyses of callus bone volume (BV) in Dnmt3bGli1ER mice and control mice 7,10, 14, 21 and 28dpf. **E** Alcian blue/Hematoxylin/Orange G (ABH/OG) staining of fracture callus sections of Dnmt3bGli1ER mice and control mice at the indicated times. Scale bar = 200 μm. **F** Histomorphometry quantification of the cartilage tissues% in ABH/OG staining at 7, 10, 14dpf. **G** Histomorphometry quantification of the bone areas in ABH/OG staining at 21, 28dpf. Data were presented as means± SEM. *P < 0.05; **P < 0.01; ***P < 0.001; *n* ≥ 4 in each group

Previously studies have identified Dnmt3b as the most regulated epigenetic factor responsive in fracture repair process. Dnmt3b expression rise and maintained in PSCs during fracture repair and gradually declined to a nondetectable level [26]. To examine the role of Dnmt3b in PSCs, we generated *Gli1-CreERT2; Dnmt3bflox/flox (Dnmt3b*^*Gli1ER*^*)* mice. *Dnmt3b*^*Gli1ER*^ mice has no obvious growth difference compared with Cre-negative mice through 10 weeks of age in body weight and size. This suggests that loss of Dnmt3b is either compensated by the presence of other Dnmts during bone development. We induced the *Dnmt3b*^*Gli1ER*^ and their littermate controls mice by intraperitoneal injection of Tamoxifen at the age of 1 month. We performed tibia fracture on 10-week-old *Dnmt3b*^*Gli1ER*^ mice and controls to determine the effect of Dnmt3b deletion on PSCs proliferation and differentiation as well as on fracture repair in mice. And them tibia was removed at 7, 10, 14, 21 and 28dpf (Fig. 1A). Micro-CT analyses of mineralized calluses in *Dnmt3b*^*Gli1ER*^ mice and control mice were showed (Fig. 1B). It showed that the callus at 10 and 14dpf is larger in control mice than in *Dnmt3b*^*Gli1ER*^ mice. Bone volume at 21 and 28dpf in *Dnmt3b*^*Gli1ER*^ mice are decreased although there was no statistical difference (Fig. 1C, D). Then, we take Alcian blue/Hematoxylin/Orange G (ABH/OG) staining to show the impaired fracture repair in *Dnmt3b*^*Gli1ER*^ mice (Fig. 1E). By 7dpf, mice had abundant chondrogenesis at the central region of fracture, an osteogenesis and intramembranous bone formation in the periosteum adjacent to the fractures site. There was no significant difference in callus size at 7dpf in *Dnmt3b*^*Gli1ER*^ mice compared with control mice. In contrast, the callus at 10 days decreased sharply and almost disappeared in *Dnmt3b*^*Gli1ER*^ mice and it last at 14dpf (Fig. 1F). The data of bone tissue was no statistical difference in preceding part at 21 and 28dpf (Fig. 1G). Taken together, our findings revealed a reduced formation of both cartilage and woven bone in the fractures of *Dnmt3b*^*Gli1ER*^ mice, suggesting that fracture healing is impaired in the absence of Dnmt3b in PSCs.

### The cell viability of chondrocytes derived from Dnmt3b ablation PSCs differentiation decreased

**Fig. 2:**
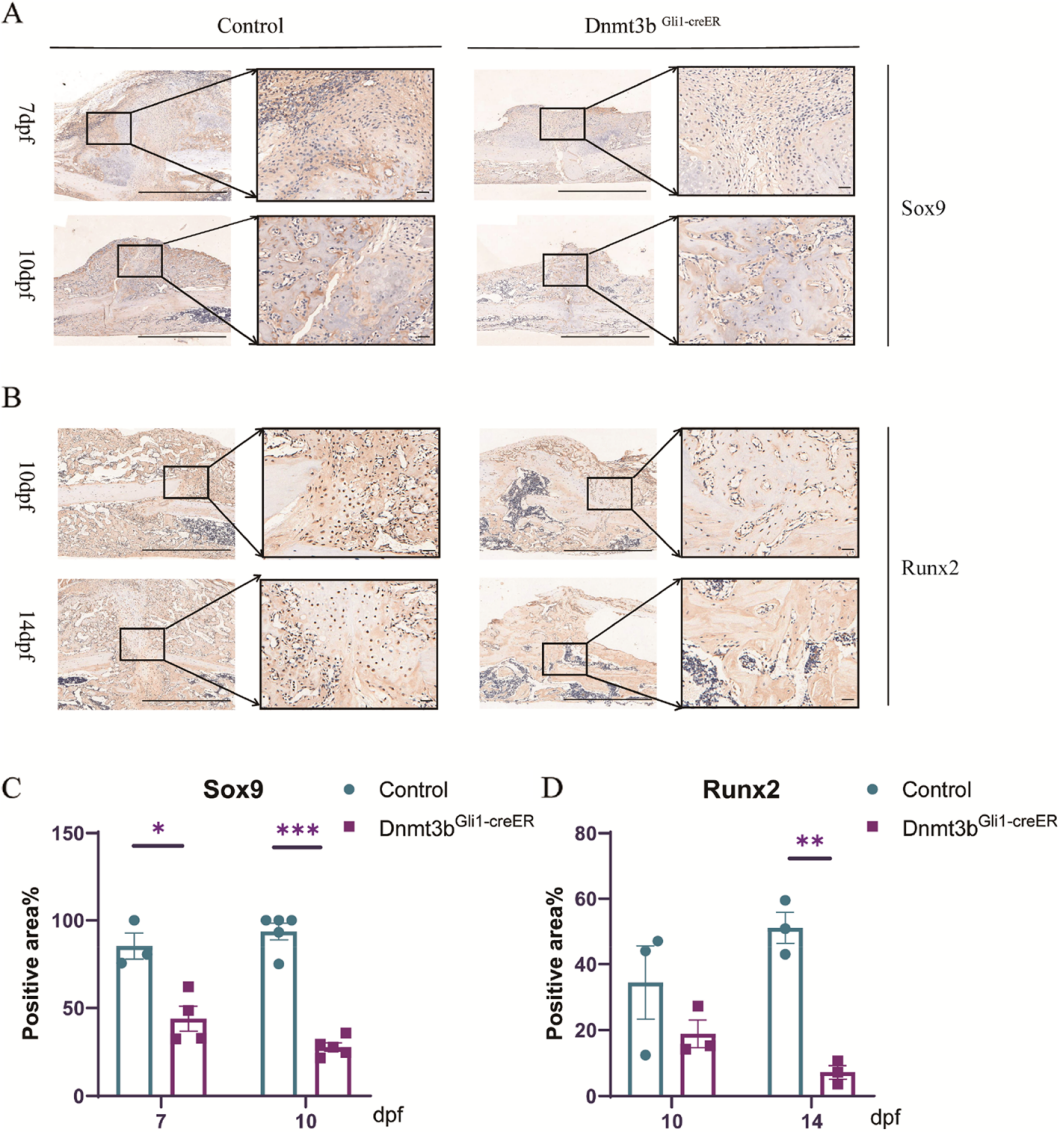
The cell viability of chondrocytes derived from Dnmt3b ablation PSCs differentiation decreased. **A-B** Expression levels of Sox9 and Runx2 genes in fracture calluses. Red arrows indicate positive cells. Scale bar = 200 μm and Scale bar = 50 μm; **C-D** Immunohistochemical staining and quantification of Sox9 and Runx2 in fracture calluses. Data were presented as means± SEM. *P < 0.05; **P < 0.01; ***P < 0.001; *n* ≥ 4 in each group

The osteogenic and chondrogenic abilities of stem cells indicate the ability of fracture healing, so we examined the chondrogenic and osteogenic indicators in the fracture healing tissues of two groups of mice. The Sox9 gene which expressed during chondrogenesis starting from the stem cell stage regulates almost all stages of stem cell differentiation such as prechondrocytes, early chondrocytes, growth plate chondrocytes, and articular cartilage chondrocytes [27]. Transcription factors (TFs) control the differentiation of chondrocytes at the level of gene expression. When stem cells differentiate into chondrocytes, some genes induce chondrocyte hypertrophy, such as Runx2 [28]. Studies have shown that affecting chondrocyte hypertrophy can lead to delayed healing of fracture [13]. So, we detected the expression of Sox9 in fracture healing tissues, the expression level of Sox9 had a decrease trend. The expression level was significantly lower at 7 and 10dpf in *Dnmt3b*^*Gli1ER*^ mice (Fig. 2A, C). Next, we detected the expression of Runx2 in fracture healing tissues, the expression level of Runx2 exactly show the same trend as sox9 at 10 and 14dpf in *Dnmt3b*^*Gli1ER*^ mice (Fig. 2B, D). In all, these results suggest that Dnmt3b knockout can cause a little decreased differentiation of stem cells to chondrocytes, more importantly the cell viability especially the ability of chondrocyte hypertrophy was impaired.

### Ablation of Dnmt3b in vivo leads to delayed bone remodeling and poor healing

**Fig. 3:**
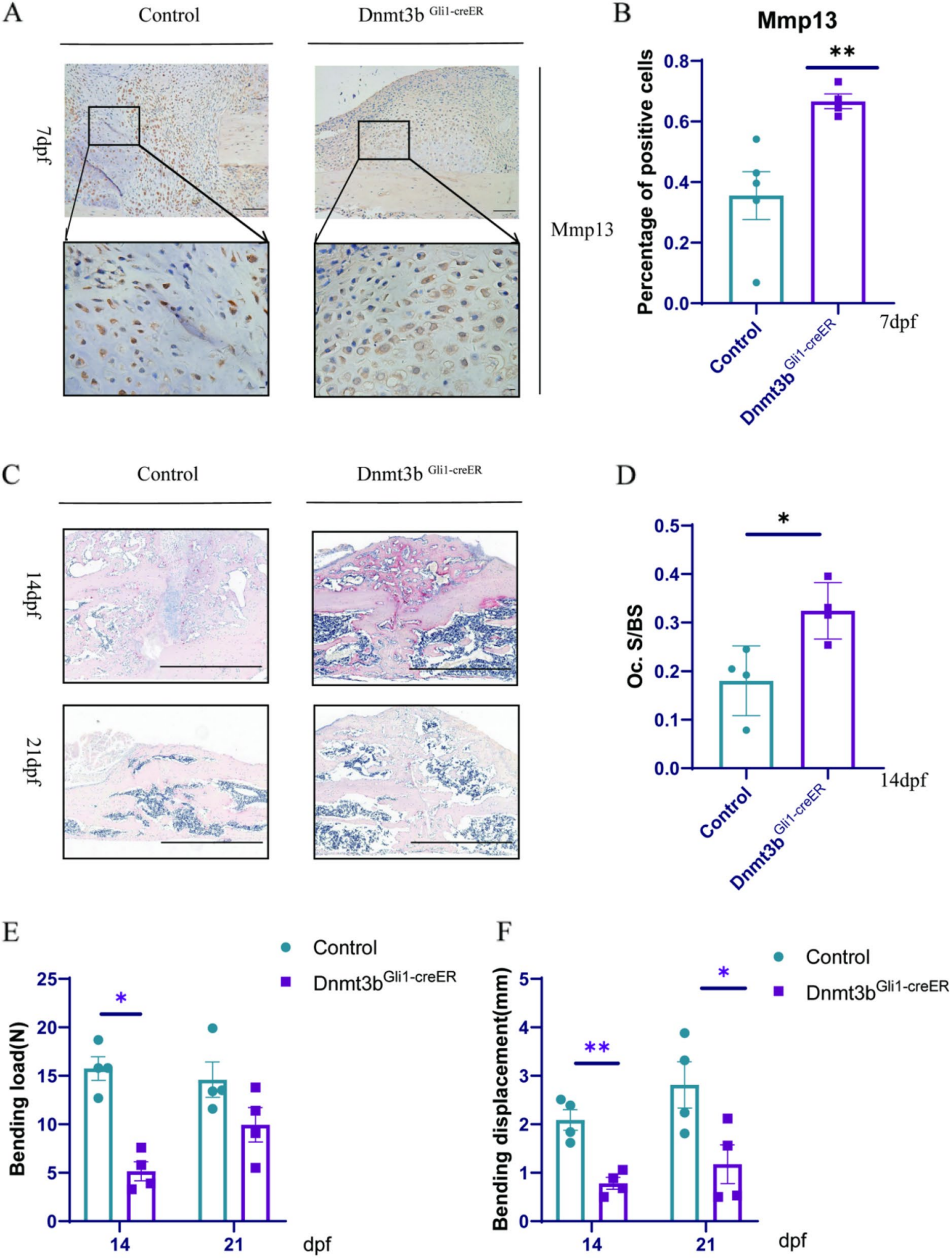
Ablation of Dnmt3b in led to delayed bone callus remodeling and decreased biomechanical properties of the newly formed bone during fracture repair. **A** Expression levels of Mmp13 genes in fracture calluses. Scale bar = 100 μm and Scale bar = 25 μm; **B** Immunohistochemical staining and quantification of Mmp13 in fracture calluses; **C** Tartrate-resistant acid phosphatase (TRAP) staining of fracture callus sections of Dnmt3bGli1ER mice and control mice at the indicated times. Scale bar = 200 μm; **D** Histomorphometry analysis of osteoclast surface per bone surface (Oc. S/BS) was performed on TRAP staining of *Dnmt3b*^*Gli1ER*^ and control fractures at the indicated times. **E** Maximum bending loading of fractured tibia 14 and 21dpf. **F** Maximum bending displacement of the fracture site 14 and 21dpf; Data were presented as means± SEM. *P < 0.05; **P < 0.01; ***P < 0.001; *n* ≥ 4 in each group

Fracture healing is an integrated process, and the formation and hypertrophy of chondrocytes and the transformation of osteoblasts are a continuous process. Mmp13 expression of hypertrophic chondrocytes in the growth plate during osteanagenesis is essential for cartilage resorption. In the fracture callus, Mmp13 is expressed by hypertrophic chondrocytes and osteoblasts, and its deficiency causes increased cartilage volume and delayed cartilage resorption in the callus [29]. So, we detected the expression of Mmp13 in fracture callus. The results showed that at 7dpf, mmp13 increased significantly in the *Dnmt3b*^*Gli1ER*^ mice, but there was not much mmp13 expression in the control group (Fig. 3A-B). The increase of osteoclasts also speeds up the bone remodeling process. So, Tartrate-resistant acid phosphatase (TRAP) staining was performed to examine osteoclast-mediated remodeling 14 and 21dpf. The total number of TRAP-positive cells peaked 14dpf in the calluses of *Dnmt3b*^*Gli1ER*^ mice and gradually decreased at 21dpf (Fig. 3C-D). More importantly, a three-point bending test was performed on fractured tibiae of *Dnmt3b*^*Gli1ER*^ mice and control mice 14 and 21dpf showed that despite comparable bone callus volume 21dpf, the *Dnmt3b*^*Gli1ER*^ mutant fractures had significantly lower mechanical properties, including bone stiffness and bone toughness compared with control mice (Fig. 3E-F). Also, there were no statistical difference with bony weight or size in the control and treated group on day 21 or 28dpf. In sum, these in vivo data show that Dnmt3b deficiency in PSCs not only impairs the formation of cartilaginous and bony callus, but also affects later events, including bone remodeling by osteoclasts and the expression of Mmp13 and the restoration of biomechanical strength.

### Dnmt3b deletion leads to the apoptosis of chondrocyte cells in the process of cartilage differentiation

**Fig. 4:**
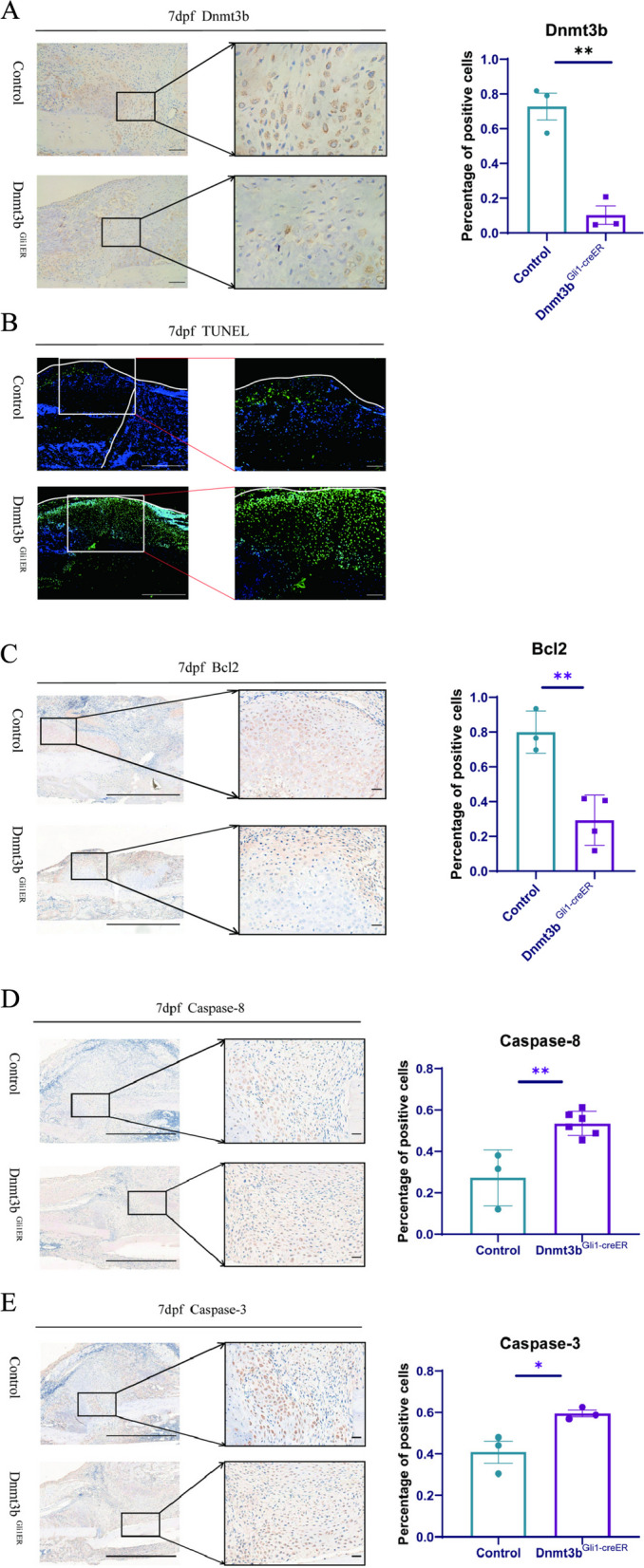
Dnmt3b deletion leads to the apoptosis of chondrocyte cells in the process of cartilage differentiation. **A** Immunohistochemical staining and quantification of Dnmt3b in fracture calluses Scale bar = 100 μm and Scale bar =25 μm; **B** TUNEL staining of chondrocytes in fracture calluses at 7dpf. Scale bar = 200 μm and Scale bar = 100 μm. **C** Immunohistochemical staining and quantification of anti-apoptotic factors, BCL-2, in fracture calluses. Scale bar = 200 μm and Scale bar = 50 μm. **D** Immunohistochemical staining and quantification of Caspase-3 in fracture calluses. Scale bar = 200 μm and Scale bar = 50 μm. **E** Immunohistochemical staining and quantification of apoptosis factors Caspase-8 in fracture calluses. Scale bar = 200 μm and Scale bar = 50 μm. Data were presented as means± SEM. *P < 0.05; **P < 0.01; ***P < 0.001; *n* ≥ 3 in each group

At 7dpf, there was no significant difference between the two groups in cartilage callus, so we examined the expression of Dnmt3b at fracture calluses at 7dpf. We found that the *Dnmt3b*^*Gli1ER*^ mice expressed almost no Dnmt3b at fracture calluses, which is consistent with our hypothesis that these chondrocytes are differentiated from which dnmt3b knockout periosteum stem cells (Fig. 4A). In order to understand how Dnmt3b affects the fracture healing process, we speculated that chondrocytes lacking Dnmt3b would trigger the apoptosis mechanism of cells. Frist, we use TUNEL staining to investigate the survival of chondrocytes in fracture calluses. Just as we expected, the percent of TUNEL positive cells in *Dnmt3b*^*Gli1ER*^ mice were more than the control group which in a certain degree indicating that Dnmt3b knockout led to chondrocyte cells apoptosis (Fig. 4B). Next, we used an anti-apoptotic protein, BCL-2, to reverify this speculation. The expression of BCL-2 was downregulated at 7dpf in *Dnmt3b*^*Gli1ER*^ mice (Fig. 4C). Furthermore, we detected the apoptosis-related factors such as Caspase-3 and Caspase-8, in fracture calluses. Both the expressions of Caspase-3 and Caspase-8 were upregulated at 7dpf in *Dnmt3b*^*Gli1ER*^ mice (Fig. 4D, E). As chondrocytes are the most abundant cells in callus healing tissue, these results suggesting that high expressions of apoptosis-related factors related transcription factors in chondrocytes might contribute to the disappearance of cartilage callus in *Dnmt3b*^*Gli1ER*^ mice.

## Discussion

Fracture is a common health problem, but the knowledge of biological processes that promote and enable fracture healing is still limited. Most fractures heal following temporary immobilization and/or surgical fixation. However, a small percentage (3–10%) of fractures fail to heal ultimately resulting in nonunion [1]. Bone fracture repair is well known as a complex and integrated series of events involving transcriptional responses unique to many cell types, including stem cells, chondrocytes, osteoblasts and osteoclasts [30]. Two models of fracture healing have been proposed, first one divide the endochondral fracture healing process into four stages, inflammation, soft callus formation, hard callus formation, and bone remodeling. Next model considers fracture healing as an anabolic phase involving chondrogenesis and osteogenesis, followed by a catabolic phase of remodeling of the cartilaginous and bony callus [6]. Both models undergo the process of differentiation of stem cells into chondrocytes, and the transition of chondrocytes to osteoblasts.

The recruitment and differentiation of endogenous progenitor cells to the fracture site is a key initial step in fracture healing. The cellular and molecular events differentiation during fracture healing have been studied in various cell and animal models [31]. But the epigenetic factors regulating regeneration are unknown. Recent epigenome screening in human fracture tissues suggests that DNA methylation has an important function in bone healing [32]. Evidence from previous studies, loss of Dnmt3b in chondrocytes decreases chondrocyte hypertrophic maturation, resulting in the persistency of unmineralized cartilage within fracture callus during skeletal repair [26]. In previous studies of knocking out Dnmt3b in Prx1-positive stem cells, they resulted in delayed fracture healing, delayed cartilage callus growth and poor fracture healing in mice [30]. Prx1-positive stem cells are generally considered to be typical mesenchymal stem cells, however Gli1-positive stem cells have been found to play an equally important role in fracture healing, it is well known that Gli1 lineage cells identifies progenitors for bone formation and fracture repair [33]. Previously reported in the literature that Gli1-positive stem cells residing immediately below the cartilage are osteogenic mesenchymal progenitors by using *Gli1-CreERT2; tdTomato* mice. Gli1 lineage cells differentiated into osteoblasts and chondrocytes during fracture healing [34]. In the meanwhile, Gli1 lineage cells have been used in many studies of bone repair and homeostasis [35, 36]. Therefore, we generated *Gli1-CreERT2; Dnmt3bflox/flox (Dnmt3b*^*Gli1ER*^*)* mice. We did tibia fracture on 10-week-old *Dnmt3b*^*Gli1ER*^ mice and controls. At 7dpf, the cartilage callus has no obvious difference in two groups. In contrast, the cartilage callus of *Dnmt3b*^*Gli1ER*^ mice sharply decline at 10dpf compared with control mice which showed in microcomputed tomography assessment and alcian blue/Hematoxylin/Orange G (ABH/OG) staining.

Our result was unexpected and inconsistent with previous results. Key transcription factors involved in chondrogenic and osteogenic cells, Sox9 and Runx2, were used to detected progenitor cell differentiation capacity. Sox9 showed a little difference in early callus formation and decreased significantly at 10dpf, while osteogenic genes showed no significant difference at 10 days and significantly decreased at 14dpf. These indicating that decreased cell viability or impaired proliferation but not progenitor cell differentiation capacity accounts for the reduced fracture callus formation in *Dnmt3b*^*Gli1ER*^ mice.

Bone remodeling is closely coupled with bone formation. In the previous report that the mice Dnmt3b knockout in chondrocyte gene and the mice Dnmt3b knockout in stem cell gene all delayed bone remodeling in fractures [24, 26]. In our study, the similarly results as they found that we saw bone remodeling changes and decreases in capacity in fractures with incremental numbers of osteoclasts in the early remodeling phase (14dpf). As the Gli1-driven gene deletion does not directly affect osteoclast precursors due to lack of Gli1 expression in these cells, the findings suggest that the reduced osteoclast differentiation is secondary to noncell autonomous signals from chondrocytes and/or osteoblasts. As is well known, Mmp13 expression of hypertrophic chondrocytes in the growth plate during osteanagenesis is essential for cartilage resorption [29]. In the fracture callus, Mmp13 is expressed by hypertrophic chondrocytes and osteoblasts, and its augment causes decreased cartilage volume and rapidly cartilage resorption in the callus. So, we detected the expression of Mmp13 in fracture callus. The results showed that at 7dpf, Mmp13 increased significantly in the *Dnmt3b*^*Gli1ER*^ mice, but there was not much Mmp13 expression in the control group. Finally, biomechanical testing, the most sensitive method to determine fracture healing, confirmed the impaired fracture healing in mice with Dnmt3b LOF in PSCs.

The cornerstone of bone homeostasis is the dynamic balance between osteoblastic bone formation and osteoclastic bone resorption. It will lead to many bone diseases such as fracture nonunion, osteoporosis and osteolysis when that balance is broken [37]. Regulated cell death (RCD) involving apoptosis is participated in the maintenance of bone homeostasis. Apoptosis plays an important role in regulating bone metabolism by affecting the activities of chondrocytes, osteoblasts and osteoclasts. Apoptosis is a type of cell death regulated which through a series of signal cascades in an orderly way. It plays an essential role in regulating growth, development, immune response and other more cellular program in organisms [38]. There are two vital pathways of apoptosis, the one is caspase-8/3 family proteins, another is Bcl-2 family proteins [39]. We first ensured that the chondrocytes in the 7dpf callus were differentiated from Gli1 cells with Dnmt3b knocked out, and then used TUNEL staining to detected apoptosis. So, we examined the expression of apoptotic genes in them. The expression of apoptosis-associated proteins all just as we expected in vivo.

Our study provides an evidence that Dnmt3b ablation can affect fracture healing and lead to poor fracture healing and the chondrocytes in the callus are derived from Gli1-positive stem cells. Also, we show that Dnmt3b ablation decreases chondrocyte hypertrophic maturation by regulating apoptosis, which further affects the process of bone remodeling.

## Methods

### Experimental animals

All animals and mice experimental procedures in this study were done in accordance with approval of the Committee on the Ethics of Animal Experiments of Zhejiang Chinese Medical University(LZ12H27001). *Dnmt3b*^*flox/flox*^ mice, originally generated by Dr. En Li, were obtained from the Mutant Mouse Regional Resource Center, Davis, CA (Cat# 29887) R26mTmGf/f [17]. In order to generate mesenchymal pro-genitor-specific Dnmt3b conditional knockout mice, *Dnmt3b* ^*flox/flox*^ mice were bred with *Gli1-CreERT2* mice (*Gli1-CreERT2* mice were donated from Rush University Medical Center). *Gli1-CreERT2;Dnmt3b*^*flox/flox*^ *(Dnmt3b*^*Gli1ER*^*)* and Cre-negative littermate controls were viable and produced in Mendelian ratios. Transgenic mice genotyping was identified by PCR with a DNA extraction kit (Sigma, St. Louis, MO, USA) from tail biopsy tissue [25]. The genptyping of the Cre allele and conditional floxed allele were described previously. *Dnmt3b*^*flox/flox*^ mice were used as Cre-negative controls. Tamoxifen (10 mg/kg body weight) was administrated daily via intraperitoneal injection to Cre-negative mice and *Dnmt3b*^*Gli1ER*^ mice for 5 consecutive days at 1-month-old in order to remove Dnmt3b alleles (Sigma, St. Louis, USA). Only male transgenic mice were used for this study to avoid gender difference confounding results on bone development and fracture healing. All mice were free for food and water in this study.

### Tibia fracture model

A unilateral transverse tibial fracture combined with intramedullary needle fixation was created unilaterally in 10-week-old male mice in our study [40]. Prior to surgery, 10-week-old mice were anesthetized with intraperitoneal injection of pentobarbital sodium (60 mg/kg body weight). After the mice were anesthetized, we performed approximate 1.8 cm long incision along the anteromedial surface of tibia to sufficiently exposure surgical region. And then we make a 25-gauge needle insert antegrade into the tibial marrow cavity through tibial plateau. Followed by temporarily withdrawn, we transected with 11-gauge surgical blade around the mid-shaft tibia. Afterwards, the 25-gauge needle which was fixation as intramedullary needle was inserted to stabilize the fractured ends. The needle was cut off within 2 mm by a wire-cutter beyond the tibial plateau. A 6–0 nylon sutures was used to closed the incision. After surgery, mice were kept in cages after recovery from anesthesia, allowing free unrestricted weight bearing, and buprenorphine was delivered up to 3 days following fractures to relieve pain. Transgenic mice were divided into two groups: the cre-negative mice group and the *Dnmt3b*^*Gli1ER*^ mice group.

### Micro-CT analyses assessment of the mineralized callus

Mice were sacrificed at days 7, 10, 14, 21 and 28 post-operation for analyses. Firstly, radiographic analysis was performed to examine the extent of fracture healing. After careful dissection and removal of the intramedullary pin, repaired tibias were imaged. Using a Micro-computed tomography (Micro-CT) (Skyscan 1176, Bruker μCT, Kontich, Gelgium) with an integration time of 300 ms, a current of 145 mA, and an energy setting of 55 kV scanned the fractured samples to analyze the bony callus formation. The threshold was chosen using 2D evaluation of several slices in the transverse anatomic plane so that mineralized callus was identified but surrounding soft tissue was excluded. Quantification for the volumes of the bony calluses was determined as previously described using the Scanco analysis software [41]. Following the initial mCT scan, the specimens were then decalcified for 2 weeks in 14% EDTA.

### Biomechanical testing

Separated bone fragments were totally connected when the 14, and 21dpf. Soft-tissue was removed from the tibia while the intact callus at the fracture site was preserved, and then removal the intramedullary nail. The sample was subjected to a three-point bending test by Axial-Torsion Fatigue Testing System (Instron, 5569R1412, USA) and data were collected by testing software (Bluehill, 2.17, USA). The proximal and distal of tibia were placed on the fixtures which were 10 mm apart and 5 mm far from the fracture site respectively. The compression load was applied at a speed of 1.5 mm/min until failure. The maximum loading and modulus of elasticity of each sample were calculated by software.

### Histology, morphometry and immunohistochemistry

The fractured tibia samples were collected at 7, 10, 14, 21, and 28dpf for detailed histological analyses. Excess muscle and soft tissue were excised. They were fixed in 4% paraformaldehyde for 3 days and decalcified in 14% EDTA (pH 7.2) solution for 2 weeks. Specimens were placed in a graded series of ethanol for dehydration and embedded in paraffin, and sectioned at a thickness at 3 μm for staining. After deparaffinage and rehydration, sections were stained using Alcian blue/hematoxylin/orange-g(ABH/OG) and tartarte resistant acid phosphatase (TRAP) in order to analyze the cartilage composition and osteoclast formation in the fracture callus tissues. Cartilage area, bone area, mesenchyme area, and osteoclast surface per bone surface (Oc.S/BS) were quantified on ABH/OG, TRAP-stained sections using the Visiopharm Integrator System. For immunohistochemical analysis, deparaffinized sections were treated with 0.3% hydrogen peroxide to quench the activity of endogenous peroxidase. The sections were then blocked for 20 min with normal goat serum (diluted 1:20) at indoor temperature. Subsequently, sections were dropwise added with primary antibodies and incubated overnight at 4 °C. Secondary anti-bodies were treated for 20 min on the next day. Positive staining was visible by using diaminobenzidine solution (Invitrogen, MD, USA). Then counterstaining was performed with hematoxylin. Immunohistochemical (IHC) staining for Dnmt3b (Abcam, ab2851), SOX9 (Abcam, ab76997), Runx2 (Abcam, ab236639), Mmp13 (ABclonal, A11755), Caspase-8 (ABclonal, A0215), Caspase-3(HUABIO, ET1608–64) and BCL2 (HUABIO, ET1702–53) were performed on paraffin sections following the antigen retrieval and colorimetric development methodologies. Non-specific IgG antibody was used as negative control. The quantification of positive staining was evaluated using Image-Pro Plus software (Media Cybernetics, Silver Spring, USA).

### TUNEL assay

To evaluate the apoptotic cells in the articular cartilage, we performed a TUNEL assay according to the manufacturer’s guideline (Beyotime, catalog C1088). Briefly, following deparaffinage and rehydration, sections were permeabilized with DNase-free Proteinase K (20 μg/mL) for 15 minutes at 37 °C. Subsequently, slides were treated with TUNEL solution and incubated at 37 °C for 1 hour in a dark environment and counterstained with DAPI for 5 minutes. Finally, TUNEL positive cells were detected by fluorescence microscope.

### Statistical analysis

All statistical analyses in this subject were performed using SPSS 25.0 software. Quantitative data from independent experiments are presented as mean ± SEM. Comparisons between two groups were made by the Dunnett ‘s t-test. Data between multiple groups were analyzed by one-way analysis of variance (ANOVA) followed by LSD test for post hoc analysis. The difference was statistically significant at *P* < 0.05.

## Data Availability

The data used to provide support for the results of this study can be obtained from the corresponding authors.
